# TEM Tomography of Pores with Application to Computational Nanoscale Flows in Nanoporous Silicon Nitride (NPN)

**DOI:** 10.3390/membranes8020026

**Published:** 2018-06-02

**Authors:** Gregory Madejski, Kilean Lucas, Flavius C. Pascut, Kevin F. Webb, James L. McGrath

**Affiliations:** 1 Department of Biomedical Engineering, University of Rochester, Rochester, NY 14627, USA; g.madejski@rochester.edu (G.M.); klucas11@ur.rochester.edu (K.L.); 2 Department of Electrical & Electronic Engineering, University of Nottingham, Nottingham NG7 2RD, UK; Flavius.Pascut@nottingham.ac.uk (F.C.P.); Kevin.Webb@nottingham.ac.uk (K.F.W.)

**Keywords:** TEM, tomography, nanomembranes, NPN

## Abstract

Silicon nanomembrane technologies (NPN, pnc-Si, and others) have been used commercially as electron microscopy (EM) substrates, and as filters with nanometer-resolution size cut-offs. Combined with EM, these materials provide a platform for catching or suspending nanoscale-size structures for analysis. Usefully, the nanomembrane itself can be manufactured to achieve a variety of nanopore topographies. The size, shapes, and surfaces of nanopores will influence transport, fouling, sieving, and electrical behavior. Electron tomography (ET) techniques used to recreate nanoscale-sized structures would provide an excellent way to capture this variation. Therefore, we modified a sample holder to accept our standardized 5.4 mm × 5.4 mm silicon nanomembrane chips and imaged NPN nanomembranes (50–100 nm thick, 10–100 nm nanopore diameters) using transmission electron microscopy (TEM). After imaging and ET reconstruction using a series of freely available tools (ImageJ, TomoJ, SEG3D2, Meshlab), we used COMSOL Multiphysics™ to simulate fluid flow inside a reconstructed nanopore. The results show flow profiles with significantly more complexity than a simple cylindrical model would predict, with regions of stagnation inside the nanopores. We expect that such tomographic reconstructions of ultrathin nanopores will be valuable in elucidating the physics that underlie the many applications of silicon nanomembranes.

## 1. Introduction

Silicon nanomembranes (e.g., porous nanocrystalline silicon (pnc-Si) and nanoporous silicon nitride (NPN) are ultrathin films (15–100 nm thick) with nanopores (10–100 nm diameters, 0.1–40% porosity) generated from the rapid crystallization of thin silicon films [1,2]. The high porosity, nanoscale thickness, and high density of nanopores enables the rapid transport of gases and liquids [3,4,5]. Nanomembranes also have excellent sieving characteristics, with the ability to discriminate between gold nanoparticles differing in diameter by only 5 nm [2]. The dominant fouling mechanism of these membranes is thought to be cake formation on the retentate side of the filter, as the internal capacity of the nanomembrane to hold foulants is miniscule [6]. Precise confirmation of foulant buildup in the nanopores is difficult. While the diameters of silicon nanomembranes are routinely measured by electron microscopy [2,7], the internal surfaces of the nanopores have proven difficult to image, requiring destructive fracture of membrane to produce cross-sections [5,8]. 

Transmission electron microscopy (TEM) is a powerful imaging method that allows for high magnification and high-resolution imaging of ultrathin samples. Structures ranging from ~100 nm down to the level of single atoms can be imaged. However, there are only a few ways to prepare samples for TEM due to the physical constraints of imaging. Samples must be sufficiently thin to transmit the electron beam (~100 nm for most preparations). Samples are typically suspended over void space, permitting unobstructed observation. This method bypasses the need for a solid substrate, which would then make transmission imaging impossible. The standard TEM substrate size is a 3.05 mm circular disc, often made of copper or silicon, with samples suspended on open grid structures. There are a variety of nanostructures that can be imaged using this technique, including nanoparticles, which are imaged on ‘lacey carbon’ modified copper grids [9]. Biological samples are typically sliced to 100 nm thick by ultramicrotomes after cryofixation and then placed on a grid substrate [10].

The ability of TEM to image nanoscale features also enables the study of nanoscale volumes. Electron tomography (ET) is a technique used for resolving three dimensional structures of nanometer-scale objects. Features of interest are tilted at a range of angles to establish depth information; as more angles are imaged, reconstructions become more precise. The resolution limit of this technique is typically between 5–20 nm with the potential to image even smaller features [11]. Most frequently paired with TEM, ET has been used to determine the structure of nanoparticles [12], gel-embedded objects [13], and biological structures [14]. As the structure of nanofeatures often governs their function, researchers have studied nanopores using electron tomography in a variety of membranes and nanomaterials as diverse as GaSb [15], α-Fe_2_O_3_ [16], nanoporous gold [17], or FIB-sculpted silicon nitride [18]. The method of manufacture will create silicon nanomembranes with different nanopore features, especially when comparing self-assembly processes [1,2,19] to direct patterning techniques [18,20]. In biological applications, the shapes and sizes of nanopores in silicon nanomembranes are particularly important in DNA sensing [18,19,21], dielectrophoretic applications [22], and ultrafiltration [8]. Many software tools have been developed to aid the reconstruction of electron tomography data, such as TomoJ [23] or IMOD [24], and these packages are often freely accessible online. Reconstructed and segmented objects can also be exported to standard file formats (STL) used in 3D printing and physical simulation software. This permits a more realistic analysis of flow behavior than can be achieved with simple cylindrical or conical models that have been used [6,7,25]. Our method of reconstruction and simulation could readily be adapted to the detailed study of electric field lines around pores, providing insights for applications such as electroosmostic pumping [26], dielectrophoresis [22], and molecular sensing [21].

Here, we use electron tomography in the analysis of NPN structures to achieve a more comprehensive understanding of the nanoscale. We then explore their simulated structure in relation to nanoscale fluidics. In this study, we first employ a custom-developed sample holder to image 5.4 × 5.4 mm^2^ nanomembrane chips at different orientations to create a ‘tilt-stack’. We then use tomographic reconstruction to extract the internal surface of the pores. Finally, we show how the reconstructed pore volumes can be put to use by importing them into computational fluid dynamics (CFD) software (COMSOL Multiphysics™, Version 5.3a) and simulating nanoscopic fluid flow through these complex structures to arrive at a better understanding of the physics of fluid flow through silicon nanomembranes.

## 2. Materials and Methods

**Figure 1:**
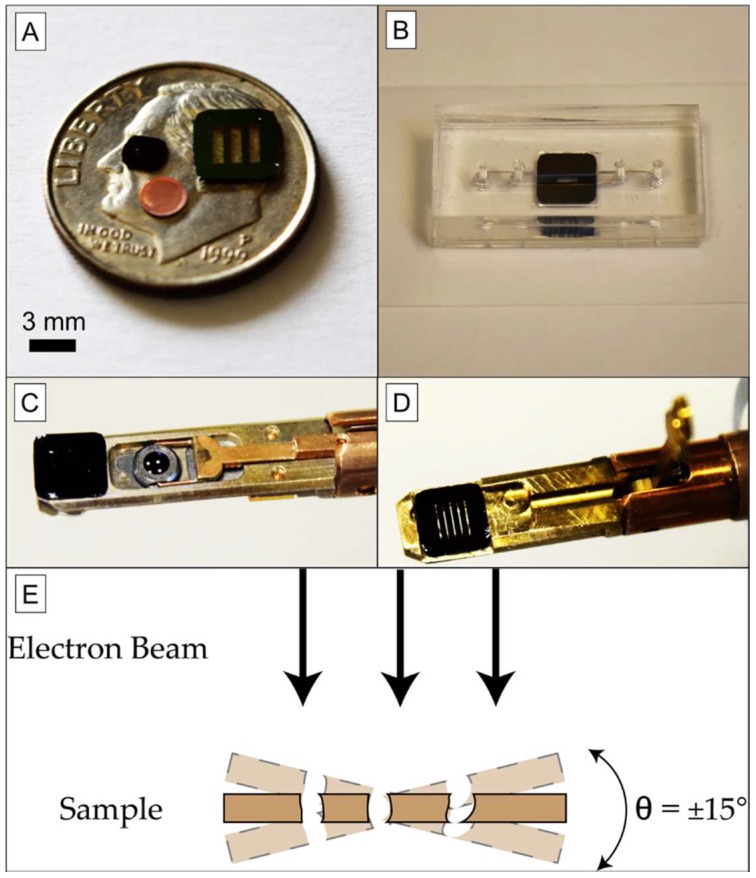
Experimental Setup. (**A**) Common TEM substrates are 3 mm circular copper grids or 3 mm silicon chips. Larger substrate formats are not commonly accepted by commercial TEM holders, since most are designed for 3 mm diameters. (**B**) A 5.4 mm square chip allows for more robust use in microfluidic devices compared to a smaller 3 mm chip or grid, which is more difficult to handle. (**C**) Image of an unmodified FEI TECNAI G20 TEM holder containing a 3-mm silicon chip, held in place by a cantilever with circular aperture (sample affixation clip), with a 5.4 × 5.4-mm^2^ NPN chip on the end for scale. (**D**) Image of a modified FEI TECNAI G20 TEM holder containing 5.4 × 5.4 mm^2^ NPN chip, with the affixation clip raised. The viewing area remains limited to the 2.8 mm circular aperture of the clip. (**E**) Tilted views of the sample are available using this modified holder, which are necessary for electron tomography.

TEM: A single tilt holder (FEI TECNAI G20 TEM, Thermo Scientific^TM^, Waltham, MA, USA) was milled to accept 5.4 mm × 5.4 mm samples by removing the stage sidewalls, leaving the sample affixation clip (Figure 1). After eucentrically centering the substrates, images were collected from nanoporous silicon nitride chips (NPN, 50–100 nm thick membranes, SiMPore Inc., Rochester, NY, USA) for ET reconstruction by tilting the chips between −14° and +14° in 2° increments. After each tilt, images were realigned using a software fiducial mark and manually moving the stage. Images were then exported into 8-bit TIF format (2048 × 2048 pixels).

Fiji/Anaglyphs: Images were imported to Fiji (https://fiji.sc/) [27], aligned using the Linear Stack Alignment with SIFT plugin (https://imagej.net/Linear_Stack_Alignment_with_SIFT), then converted to 8-bit two-shot RGB anaglyphs from the original TIF using an open-source plugin (https://imagej.nih.gov/ij/plugins/anaglyph.html) for 3D display. 

ImageJ/TOMOJ: For ET reconstruction, three sets of original tilt-stack images were imported into ImageJ (https://imagej.nih.gov/ij/download.html) [28], scaled to 400 × 400 pixels, and sorted in order from negative angle to positive tilt angle within each stack. Using the TomoJ plugin (v2.35, http://www.cmib.fr/en/download/softwares/TomoJ.html) [23], ET tomograms were generated from each tilt-stack. The stacks were preprocessed and aligned within TomoJ before reconstruction. Blank pixels were filled with the average value of the image. Hot spots were removed at a radius of 3 pixels. The ordered stacks were background-corrected using a 50-pixel sliding parabola (rolling ball, light background). Stacks were pre-aligned by shifting the center of mass in each image using a cross-correlation translation correction. Landmarks were generated (200 seeds, 5 minimum chain length, 20 pixel patch size), then a landmarks-based 3D alignment was performed by mapping local minima (listed as ‘new version’ algorithm) [29]. Tomograms were computed using the OS-SART algorithm (30 iterations, 0.1 relaxation coefficient, 300 pixel thickness) [30]. Estimates of surface roughness were obtained by manually cropping tomograms to the nanopore walls and use of a surface roughness plugin (https://imagej.nih.gov/ij/plugins/roughness.html).

SEG3D2: Tomograms were imported into the SEG3D2 software package (v2.4.2, http://www.sci.utah.edu/cibc-software/seg3d.html) [31]. They were then cropped from x and z projections to the thickness of the nanomembrane, and then intensity corrected (2 polynomial order, 0.15 edge sensitivity). Individual nanopores were identified using the neighborhood-connected filter, with 5–10 seeds placed on the feature of interest. As each individual feature was identified, seeds were cleared, and another feature was then identified by placing seeds on the new feature and running the filter again. Once all features were identified, layers were merged into a single segmentation layer using Boolean OR filters. Noise in the segmentation layer was removed by using a smooth binary dilate and erode filter (value = 2) or a 2D fast binary dilate and erode filter (value = 6). Small openings in incompletely etched nanomembranes were identified manually at the bottom orifice. Isosurfaces were then computed and exported to an STL file format. Line sections were taken of nanopore sidewalls along XZ and YZ projections for smoothed and unsmoothed contours, and the RMS roughness (*R*_q_) was calculated based on a linear regression of the pore wall (Supplemental Section S7).

Meshlab: STL files generated from the above segmentation were re-meshed using Meshlab (v2016.12. http://www.meshlab.net/) [32] into ~1000 faces/nanopore, using a quadric edge collapse decimation filter with the topology preservation option selected. Simplifying the mesh to contain fewer faces facilitated an import into COMSOL Multiphysics™ with significantly fewer geometry errors.

COMSOL Multiphysics™: STL files were imported into COMSOL Multiphysics™ (Version 5.3a, https://www.comsol.com/) using the mesh import feature. Minimal boundary recognition was used during the import to reduce the number of non-physical boundaries generated by the software. After import, the mesh was converted to a geometry object. Faulty features were identified by the software upon import and manually deleted. The desired pores were then converted to solid objects. To enable flow through the pore features, blocks were created to overlap the top and bottom of the pores by 3 units, making a clean boundary. These boundaries were defined by partitioning the pore with these block objects (Supplemental Section S4). The domain was defined to contain water (1 atm, 20 °C) and then the Laminar Flow physics package was added to the simulation. The larger pore orifice was set as the inlet, with a velocity boundary condition, and the smaller orifice was set as the outlet, with a zero-gauge pressure condition. The geometry was meshed using a normal mesh and a stationary study was performed using a nonlinear PARADISO solver configuration. Error tolerance was set to default (convergence at error <0.001) and then the study was computed. Results were plotted as surfaces and as a two-dimensional slice through the *yz*-axis to generate a velocity profile through the pore axis.

## 3. Results

A FEI TECNAI G20 sample holder was modified to accept larger area NPN chips. Common TEM substrates are displayed in Figure 1A on the face of a dime, as circular 3 mm substrates. A 5.4 mm square chip containing much larger free-standing NPN windows (SiMPore Inc.) is also shown; the windows are transparent due to their thinness. These chips can be used in microfluidics and other applications that would be useful in capturing analytes for observation. The 5.4 mm square format provides a more robust platform than the 3 mm substrate for any microfluidic application, allowing for simple device design (Figure 1B). As the standard TEM holder (Figure 1C) is 5.6 mm wide, it can support the larger 5.4 mm square chips after careful milling of the side walls, while the sample affixation clip is preserved to retain the 5.4 mm sample (Figure 1D). The sample holder can then be used in normal operation (Figure 1E). This modification allows larger samples to be prepared for TEM imaging, so long as the sample area of interest can be aligned to the central 2.8 mm observation ring.

**Figure 2:**
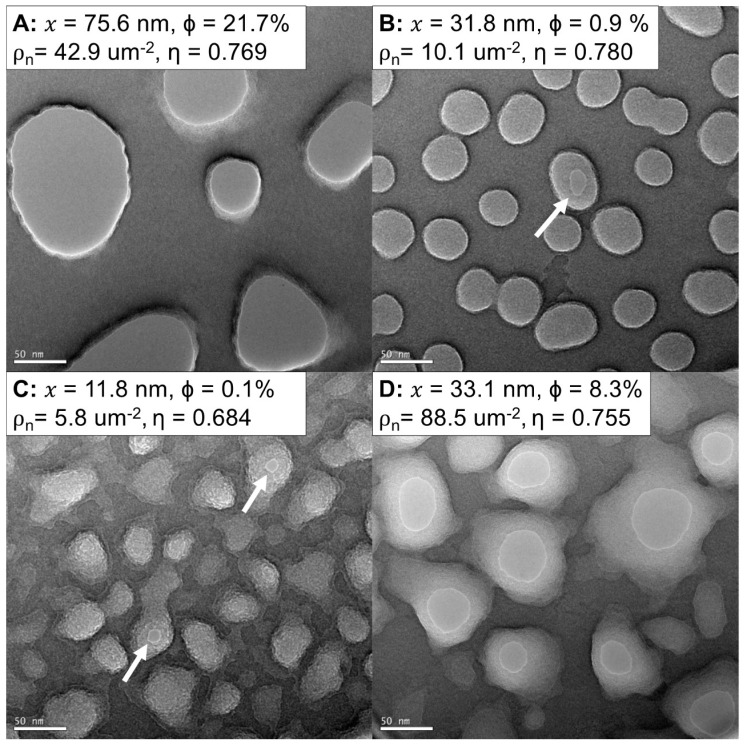
NPN membranes manufactured with different nanomorphologies. (**A**–**D**) TEM images (63 kx, 0°) of four different varieties of NPN. The sizes and shapes of the pores can range from largely open (**A**,**D**) to pitted and under-etched (**B**,**C**). White arrows indicate open nanopores in low-porosity films. Parameters derived from histograms of counted pores (Supplemental Section S1) show the wide variety of membrane porosities and pore diameters. *x* = Average Pore Diameter, Φ = Porosity, ρ_n_ = Pore Density, η = Circularity.

Figure 2 displays samples of NPN on 5.4 mm square chips imaged with TEM. Nanomembrane properties are listed as averages of many pores generated from lower-magnification (9–17 kx) images (Supplemental Section S1). The manufacture of the nanopores in the NPN chips creates features that are wider on the flat surface of the chip (top) compared to the trench-facing surface (bottom) [1]. Nanopores were thresholded to yield the smallest orifice at the bottom of the nanopore. A wide variety of nanotopographies and pore shapes can be created by the NPN fabrication process [1]. While many of the open pores appear to have straight sidewalls, some pores taper to more uniform ellipses (10–30 nm) from much larger (50–80 nm) anisotropic openings. The thickness of the nanomembrane means that many of these pores have an aspect ratio approaching 1:1, but the tapering of the pore adds additional geometry likely to be consequential to the functional properties of the membranes. Under-etched membranes appear pitted (Figure 2B,C), as these pores did not fully etch into the silicon nitride film, thus leaving membranes with significantly reduced porosity.

**Figure 3:**
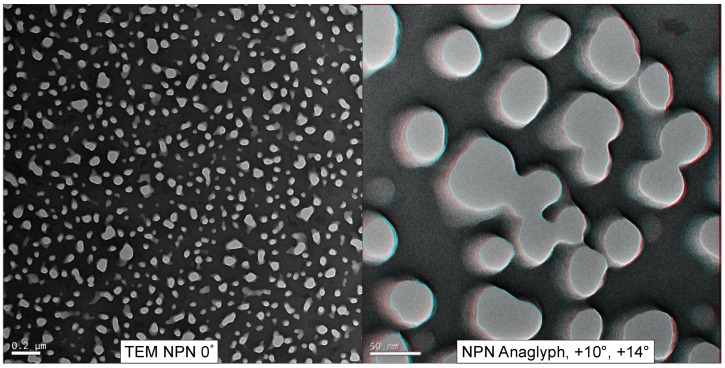
NPN Anaglyph. NPN (50–100 nm thick) was imaged from −14° to +14°, in 2° increments. The anaglyph was formed by taking the +10° and +14° images, aligning them using Fiji [27], and assigning them to red and cyan color channels. Viewed with red/cyan colored glasses, the anaglyph can provide the illusion of depth (best viewed on a computer screen).

Figure 3 displays a low-magnification TEM view of an NPN membrane (*en face*) as well as a red/cyan false-colored anaglyph of NPN nanopores created from a tilt-stack. The red and cyan channels are images gathered at 4° angular separation. By emulating the binocular offset of human pupils (~6 cm, producing ∼3–4° angular separation at 10 cm of depth) a 3D visual illusion (stereopsis) is created while wearing red/cyan glasses, since each colored image is sent to a different eye. The nanopores displayed in Figure 3 depict irregular contours and smooth sidewalls within a nanomembrane that is 50 nm thick.

While stereoscopic images are useful to visualize the nanotopography of the NPN, they are reliant on our own visual processing system to ascribe depth to features within the image. Information from tilted images here permits the quantitative reconstruction of depth using ET. In this work, NPN nanomembranes were imaged using a series of tilts from −14° to +14°, in 2° increments (2048 × 2048 pixels). While this is a narrower range of angles than is typically used in ET, it is more than sufficient to extract the internal contours of narrowing nanopores. The scale of nanofeatures in these images (10–50 nm) does not require atomistic resolution and can be represented with ~1 nm/pixel resolution (imaged at 0.17 nm/pixel).

**Figure 4:**
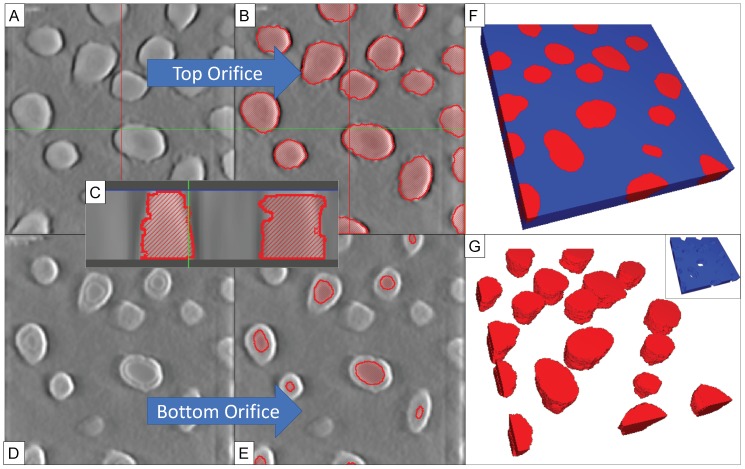
Method for Generating 3D models from Tilt Stacks. (**A**) As tomograms can be generated from the tilted images; (**B**) individual structures can be segmented at different layers within the stack by regions of similar intensity; (**C**) A cross section of the reconstruction highlights the nanopore walls; (**D**) Reconstructed layers can merge the bottom orifice with a thinly floored pore due to similar intensities; (**E**) requiring manual adjustment to the narrowest orifice; (**F**) Segmented nanopores in a volume stack, viewed on the largest orifice; (**G**) Reconstructed nanopores (red) or the nanomembrane (blue inset, inverse of **G**) can then be exported into other software for visualization or simulation purposes.

Figure 4 describes a method for generating a 3D model from these tilted images. TomoJ [23], an ImageJ [28] plugin, was used to map the tilted images into a gray-scale height stack using an OS-SART algorithm [30]. The reconstructions of native images (2048 × 2048 pixels) can be large (5 GB) and very time consuming (>4 h), therefore images were scaled down to 400 × 400 pixels before tomogram computation, producing ∼200–300 MB sized reconstructions in minutes. The reconstructions were cropped along the *z*-axis to feature only the nanomembrane (~55–110 pixels, 0.89 nm/pixel). Each of the contours in the tomogram was then segmented into different nanopores, using SEG3D2 [31] segmentation software (Figure 4B). Assigning segments to different heights in the stack and knowing the physical dimension of the NPN thickness allows us to characterize the morphology and location of different contours in the nanopore. There are many ways to identify the body of the nanopore; the pores shown here were identified by regions of similar intensity. In Figure 4C, the difference between a nanopore and a pit is highlighted in cross-section. An estimate of the surface roughness along the segmented pore sidewalls is higher than observed in the raw tomogram (*R*_q_ = 1.52 nm vs. *R*_q_ = 0.72 nm, Supplemental Section S7). Incomplete erode/dilation artifacts are responsible for the increase in roughness, though other smoothing during post-processing of isosurfaces can lower this roughness further (*R*_q_ = 1.05 nm). As such, the segmentations are overestimating the roughness of the pore walls; TEM images show smoother sidewalls. As the bottoms of incompletely etched nanopores can be very thin (Figure 2B and Figure 4D), some of the pitted contours do not provide sufficient contrast for automatic segmentation in reconstruction and are thus manually adjusted to the narrowest orifice (Figure 4E). While the bottom orifice constrictions are clear, the regions of poor contrast create uncertainty in the thicknesses of these pore floors, due to the resolution of the reconstruction (*d_z_* = 1.8 nm, Supplemental Section S2); we have adjusted up to 5 contours along the bottom orifice to interpolate this uncertainty. As the pore volumes are segmented, the inverse of the pores (nanomembrane body) can be easily extracted using a Boolean subtraction (Figure 4F). These pore volumes can be exported directly as isosurfaces (Figure 4G), or the nanomembrane body itself (Figure 4G inset). The finalized contours were then exported into a 3D STL file, which can then be imported into other programs for simulation or visualization purposes, including tangible 3D-printable models (Supplemental Section S3).

**Figure 5:**
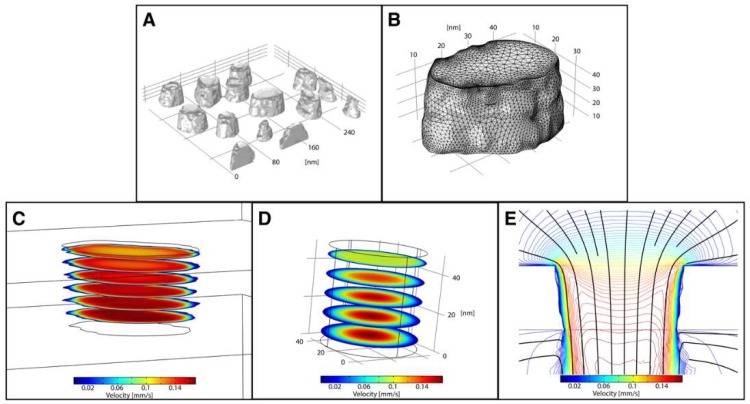
COMSOL Multiphysics™ Simulations of Nanopores Reconstructed by Electron Tomography. (**A**) Reconstructed nanopore STLs can be representative of a region of a membrane with multiple pores or (**B**) of a single pore, where both have nanoscale structure. (**C**) Fluid flow simulations of B, showing regions of irregularity along nanopore sidewalls. (**D**) The velocity profile of a simple cylindrical representation of the nanopore in B. (**E**) Velocity contour plot of C, with streamlines overlaid (black). Flow enters from the top visible plane of the pore at *V* = 5.7 × 10^−5^ m·s^−1^ and exits at the bottom plane at atmospheric pressure (*P*_gauge_ = 0). (**B**,**C**,**E**) are representations of the same nanopore at the same scale.

Figure 5 shows the results of a COMSOL Multiphysics™ simulation where the tomographically generated STL defined the system geometry. Using the mesh import option, we can create geometries with multiple pores (Figure 5A), allowing for multi-pore analysis without making generalizations of nanopore structure. However, STL files with large numbers of faces caused meshing errors during their import into COMSOL, so the raw STL files were re-meshed using Meshlab [32]. Single pores can be analyzed at the nanoscale with a higher density computational mesh (Figure 5B) to create a more accurate solution set. We were able to import both simple, single channel pores (Figure 5) as well as more complicated, bifurcated pore geometries (Supplemental Section S5), capturing the variety of nanopore structures that occur within NPN.

We then applied a laminar flow simulation to the system to compare fluid flow in a more complex geometry against a simple cylindrical pore representation. Assuming a flow rate through a 2.0 mm × 0.7 mm membrane (~3.0 × 10^8^ pores) of 10 μL·min^−1^, and an average pore diameter of 50 nm, we set the inlet velocity approaching each pore to 57 µm·s^−1^. These characteristics are representative of flow conditions that are commonly used in microfluidics with these nanomembranes [33]. This flow was laminar (*R*_e_ = 2.9 × 10^−6^), supporting a crucial underlying assumption of the chosen physics package (Supplemental Section S6). The outlet of the membrane was assumed to be open to the atmosphere (zero-gauge pressure, *P*_gauge_ = 0).

The results of the simulation show how the complexity of the pore volume influenced the flow (Figure 5). In addition to the expected low-velocity flow regime near the pore wall, due to the no-slip condition, the nanostructured surface appears to create pockets where these regions extend several nanometers from the wall (Figure 5C). These regions would likely be susceptible to fouling by protein or particulate adhesion. The development of the boundary layer through the pore is also more complex in the reconstructed pore as compared to a simplified, cylindrical pore model (Figure 5D), where the boundary layer is axisymmetric. As the fluid moves through the reconstructed pore, the boundary layer is thin at the entrance orifice, and the flow profile becomes increasingly parabolic (Figure 5E). However, the profile becomes blunter approximately half-way through the pore, as the influence of the exit (uniform pressure boundary condition) interrupts further development of parabolic flow.

## 4. Discussion

In this study, we have used ET to establish the 3D morphology of NPN nanomembranes and used the resulting data to simulate the effects of 3D nanofeature geometry on fluidic behavior at the single- to multi-pore scale. Instead of imaging single pores, we can image and reconstruct a large number of nanopores in the same field of view, due to the high density of features in NPN. This is particularly valuable because the self-assembled nanopore formation inherent to the pnc-Si/NPN templating process can produce a diversity of pore shapes and sizes within the same membrane. After acquiring a tilt-stack image sequence on 20 standardized 5.4 mm square NPN substrates (0.01–1.4 mm^2^ window sizes, 50–100 nm thicknesses), we used freely available software to manually identify regions of interest to be segmented in a subset of these stacks, and then generated pore volumes by identifying regions of similar intensity. The structure of NPN facilitates our ability to do this reconstruction because it is ultra-thin (50–100 nm) and has smooth membrane faces, thus unambiguously defining the orifices of the pores.

The current study has suggested a new approach to codifying nanopore nanostructure, and its potential significance to nanoscale fluidics within these pores. The resulting improved physical simulations demonstrate the benefit of tomographically reconstructing nanopore geometries to better understand nanoscale phenomena that occur within a pore. Typically, simplified representations (e.g., cylinders, cones) of complex geometries are used in simulation software to quickly predict system behavior. However, COMSOL allows users to import complex and accurate geometries for more comprehensive analyses. Regions of stagnation and complex internal flows are revealed by simulating nanoscale fluid flow through the reconstructed contours of real nanopores. As fluid moves through the pore, the boundary layer begins to thin, and the flow profile becomes increasingly parabolic (Figure 5E). Interestingly, the velocity profile appears blunted in the lower part of the pore as it approaches a uniform pressure at the exit orifice. Thus, flow never fully develops in the nanopore because exit effects influence a significant portion of transport through the pore. Other work has been performed for the analysis of track-etched filter membranes, where pores typically have a 10:1 length to diameter ratio [34,35]. These higher-aspect-ratio pores allow for the fluid flow to fully develop within the pore, which never occurs within a nanomembrane pore in our simulations. In agreement, simulations of electroosmotic flow in nanopores with 1:1 length to diameter ratio have been shown to exhibit non-uniform flows due to flow expansion or contraction at the ends of pores [36]. 

Examining the tomographically generated geometry and the simple cylindrical geometry, it is clear that the development of the fluid boundary layer is influenced by more than the pore’s aspect ratio. Convective transport processes across nanopores are assumed to have ballistic trajectories [5,7,8], but the low-flow regimes within these nanopores may present opportunities for adsorption and pore-wall fouling, as seen with diffusive transport [2]. Currently, cake formation on the retentate side of the nanomembrane is considered to be the dominant mode of fouling with nanoscale membranes, since the internal volume of each nanopore is very small due to the membrane thinness [8]. If a population of irregularly shaped nanopores also facilitates fouling by constriction or internal stagnation, this may contribute to quicker cake formation by reducing permeability. This knowledge can then be applied to improve filtration, capturing and sensing applications with these geometries.

## 5. Conclusions

In this work, we have developed a method for the tomographic reconstruction of pores in nanoporous silicon nitride (NPN). These pores are irregular in shape because of the self-assembly of pores in the template structure [2], and a transfer process into silicon nitride film that creates additional complexity such as merged pores [1]. Our reconstructions were created from tilted images from a FEI TECNAI G20 TEM (−14°~+14°, 2° increments) and freely available electron tomography software (ImageJ, TomoJ, SEG3D2, Meshlab). We then illustrated how these reconstructions can be used to improve our understanding of fluid flow profiles within nanomembrane pores by direct import and simulation in COMSOL Multiphysics™. A similar approach could be used in the future to predict electric field contours around nanomembranes [22], as well as fouling analysis for alternative nanomembrane structures (graphene-oxide mat [37], silicon sheet [38], or anodized alumina [39]). We anticipate that these techniques will prove useful for improving our understanding of electrical and transport phenomena in a host of applications involving silicon nanomembranes.

## Supplementary Materials

The following are available online at http://www.mdpi.com/2077-0375/8/2/26/s1, Figure S1: Example nanopore statistics. Histograms of nanopore properties were generated from background corrected, thresholded TEM images of NPN membranes (red pore outlines). The pores on the outer edge of the image were omitted (green pore outlines). The green square indicates the size of the background correction, averaging over many nanopore areas, Figure S2: Manually thresholded nanopores. Pores are tapered from largest orifice to smallest orifice in simulations, Figure S3: Example FSC curve generated from tomogram, Figure S4: Reconstructed NPN Nanomembrane from Electron Tomography. (A) Example printed on Makerbot Replicator 2X. (B) Example rendering of NPN model (Blender 2.79b), Figure S5: COMSOL Multiphysics™ operations for partitioning imported STL geometries. (A) An imported raw geometry converted to a solid object in COMSOL does not contain clearly defined boundaries. By partitioning the geometry (B) with planar partition objects, we define clean entry and exit boundaries. (C) Using two block structures that overlap the partition points, we can perform a Difference operation (D) to create flat surfaces free of import errors, and thus wieldable for physics definitions, Figure S6: Example Bifurcated Pore Imported for COMSOL Multiphysics™ Simulations. (A) More complex geometries can be imported into COMSOL, such as bifurcated pores. The etching process caused pores that were in close enough proximity to merge at their inlet, creating a single entrance orifice. (B) Fluidic simulation of A, showing higher velocity in the shallow bridge between pores as well as the faster velocity in the constrictions downstream. (C) Velocity surface plot of A superimposed with streamlines, Figure S7: Overlaid 20 projected contours of a segmented nanopore.
