# Supplementary material for: TEM Tomography of Pores with Application to Computational Nanoscale Flows in Nanoporous Silicon Nitride (NPN)
Source: Membranes (Basel). 2018 Jun 2;8(2):26. doi: 10.3390/membranes8020026 (PMC6027491; doi:10.3390/membranes8020026)
Supplement: Supplementary file 1 [file membranes-08-00026-s001.docx]

Supplementary Materials: TEM Tomography of Pores with Application to Computational Nanoscale Flows in Nanoporous Silicon Nitride (NPN)

Gregory Madejski ^1^, Kilean Lucas ^1^, Flavius C. Pascut ^2^, Kevin F. Webb ^2^ and James L. McGrath ^1,^*

^1^ Department of Biomedical Engineering, University of Rochester, Rochester, NY 14627, USA; g.madejski@rochester.edu (G.M.); klucas11@ur.rochester.edu (K.L.)

^2^ School of Electrical & Electronic Engineering, University of Nottingham, Nottingham NG7 2RD, UK; Flavius.Pascut@nottingham.ac.uk (F.C.P.); Kevin.Webb@nottingham.ac.uk (K.F.W.)

***** Correspondence: jmcgrath@bme.rochester.edu; Tel.: +1-585-273-5489

Section S1: Nanopore Statistic Generation

Nanopore statistics were generated using a custom MATLAB pore processing software [40]. TEM images were cropped, background corrected, and thresholded to generate open pore shapes. Pores were dilated twice and then eroded twice to merge edges, and then recorded. Aberrant pores were removed manually. Pore diameters were calculated using two methods: 1. Converting the thresholded nanopore area into an equivalent circular diameter, or 2. Establishing an ellipse based on the major/minor axes of the thresholded nanopore area and calculating an equivalent circular diameter from the ellipse.

**Figure S1.** Example nanopore statistics. Histograms of nanopore properties were generated from background corrected; thresholded TEM images of NPN membranes (red pore outlines). The pores on the outer edge of the image were omitted (green pore outlines). The green square indicates the size of the background correction; averaging over many nanopore areas.

As an alternative method of thresholding, nanopores can be manually painted to identify both the top and bottom orifices in the nanomembrane. The top orifice of NPN will nearly always be larger, due to the additional reactive ion etch step during fabrication.

**Figure S2.** Manually-thresholded nanopores. Pores are tapered from largest orifice to smallest orifice in simulations.

Section S2: Reconstruction Resolution Estimation

It is desirable to use a full range of angles in reconstruction. Most commonly, +/− 60–70^°^ is quoted as an acceptable range due to the restrictions of the holder [14]. As more angular information is gathered, the more accurate the depth profiles of the reconstructions will be. The single-tilt TEM holder modified in the paper has a usable range of −14° to +14°. In an ideal case, the Crowther criterion [41], gives the number of views (N) necessary to reconstruct a spherical particle (diameter D), with resolution (*d* = 1/*R*_max_) in evenly-spaced angular increments (Δα, degrees):

| $N=\frac{\pi D}{d}$ | (1) |
| --- | --- |

Rearranging for the resolution (*R*_c_ = *d*)

| $R_{c}=\frac{\pi D}{N}$ | (2) |
| --- | --- |

If we were able to gain the whole −90° to +90° range, assuming a 50 nm diameter particle and 2° increments (*N* = 90 images), *R*_c_ = 1.74 nm. With *N* = 15 images over this range, our structural resolution becomes *R*_c_ = 10.5 nm, a factor of 5 less than the thickness of our thinnest membrane. In our case, we are limited to -14° to +14°, and our feature of interest is not spherical, rather more like a slab. The resolution for a slab of reconstructed thickness *T* is [42]:

| $d_{z}=\frac{\pi(\Delta\alpha) T}{180 \cos(\alpha_{max})}$ | (3) |
| --- | --- |

For our imaging conditions (14° maximum angle, 2° increments, 50 nm thick membrane), *d_z_* = 1.80 nm, which is sufficient to capture variation in a 50 nm thick nanomembrane. An elongation artifact will occur as we do not have all of the information from the full set of rotations. An estimate of this elongation factor (*η*), is given as [14]:

| $\eta=\sqrt{\frac{\alpha+cos(\alpha)sin(\alpha)}{\alpha-sin(\alpha)(cos(\alpha)}}$ | (4) |
| --- | --- |

For *α* = 14°, the elongation correction factor is 7.06. The reconstruction algorithm used here is OS-SART, which is not as sensitive to this elongation compared to other algorithms (such as WBP, stated in the TOMOJ manual). A larger range of angles minimizes this elongation correction (*α* = 70 degrees, *η* = 1.3), as well as minimizes the information from the wedge of unimaged angles. Sharply contrasted orifices with smaller sizes (2–3 nm thick rings) also improve the localization of the tomogram cropping for segmentation.

Others have estimated the resolution of reconstructions from SIRT and ART algorithms using a Fourier-shell correlation (FSC) metric below 0.143 or 0.5 [14]. This function is available in TomoJ. With a sample reconstruction, our Nyquist frequency for the 400 × 400 pixel reconstruction is 2.3 nm^−1^
(1.15 pixel/nm). This gives the frequency for a FSC response level of 0.5 as 0.44 nm^-1^, or the frequency for a FSC response of 0.143 as 0.67 nm^−1^.

**Figure S3**: Example FSC curve generated from tomogram

Section S3: Example 3D model Applications

STL files generated from the electron tomography reconstructions can be directly imported into Makerbot Print software (v2.8, https://www.makerbot.com/download-print/) for use with Makerbot, or other desktop 3D printers. After rescaling the object to an appropriate thickness to highlight both contours and thickness of the nanomembrane, the models were printed in ABS (220 °C, 300 μm layer thickness) on the heated bed of a Makerbot Replicator 2X. STL files can also be imported into a variety of visualization and animation software (e.g. Blender v2.79b, www.blender.org), for better examinations of cross-sections.

**Figure S4.** Reconstructed NPN Nanomembrane from Electron Tomography. (**A**) Example printed on Makerbot Replicator 2X. (**B**) Example rendering of NPN model (Blender 2.79b)

Section S4: COMSOL Partitioning Method

Partitioning the imported geometry in COMSOL is important for successful simulations. The import feature causes small errors at the top and bottom of the pores that prevent proper union operations from being performed when trying to create a whole geometry. Additionally, because we selected minimal boundary definition, the geometry will be a single object without faces to define as inlets or outlets (Figure S5A). To partition the geometry, two blocks were created and defined as partition objects under the *Partition Objects* operation in COMSOL. These blocks were created to overlap the top and bottom of the geometry by 3 units to eliminate import errors near the object surfaces (Figure S5B). With the partition planes defined, additional blocks were created in the same spot as the partition blocks (Figure S5C). A *Difference* operation was then performed to remove the top and bottom surfaces, creating flat faces, corresponding to the flat external membrane faces, that could then be defined as boundaries for physics simulations (Figure S5D).


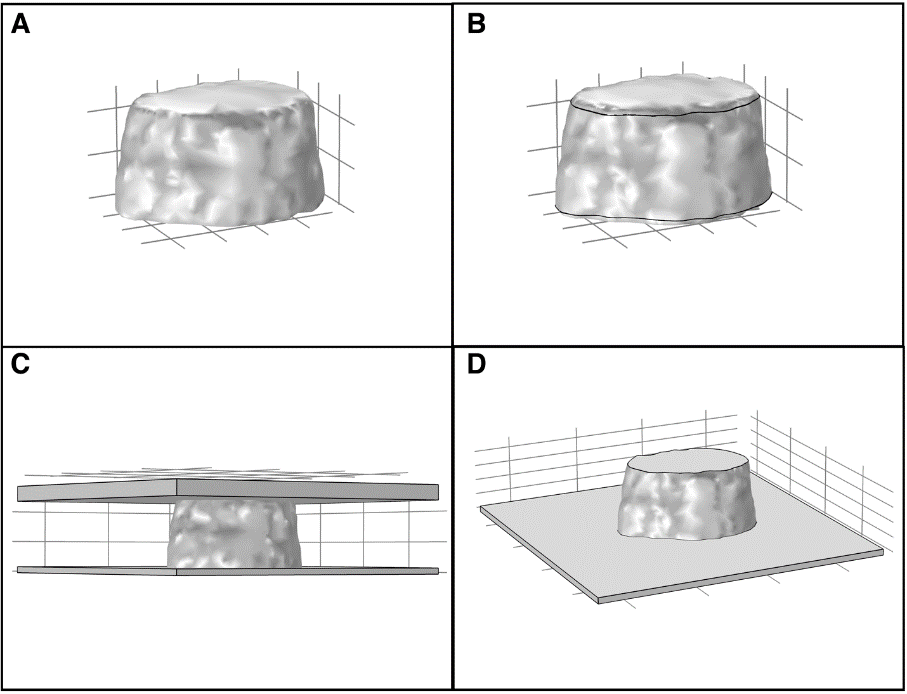


**Figure S5:** COMSOL Multiphysics™ operations for partitioning imported STL geometries. (**A**) An imported raw geometry converted to a solid object in COMSOL does not contain clearly-defined boundaries. By partitioning the geometry (**B**) with planar partition objects, we define clean entry and exit boundaries. (**C**) Using two block structures that overlap the partition points, we can perform a Difference operation (**D**) to create flat surfaces free of import errors, and thus wieldable for physics definitions.

**Section S5: Bifurcated Pore Simulation**

In addition to single pore geometries, we were also successful in importing more complex geometries, such as a bifurcated pore (Figure D1). These are not simulations that could easily be performed with simplified cylindrical geometries, and thus provides a powerful demonstration of the versatility of our technique. The COMSOL simulation of this pore permits the observation of how the fluid flow behaves within each structure, as well as at the junction between the two pores. Boundary conditions are defined as in Figure 5, with flow moving from top to bottom. High speeds occur over the septum partitioning the inlets to the two pores, and again at the narrowed outlets.


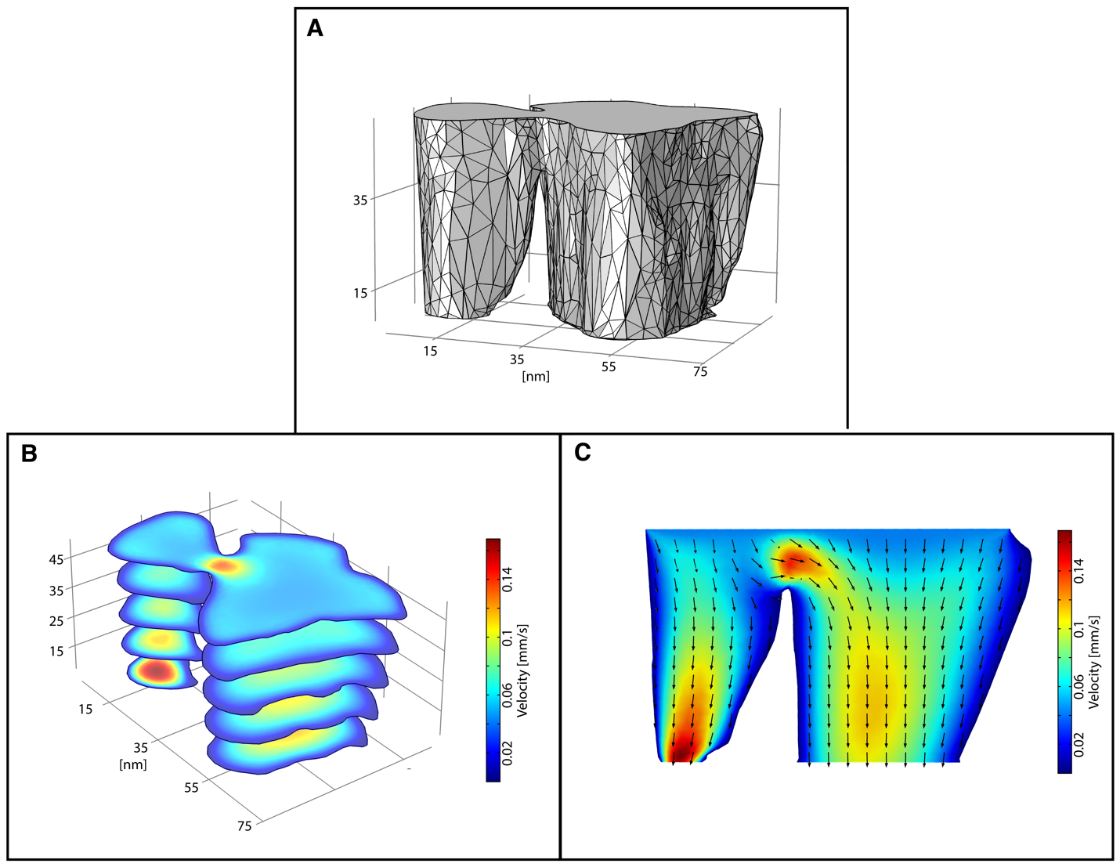


**Figure S6.** Example Bifurcated Pore Imported for COMSOL Multiphysics™ Simulations. (**A**) More complex geometries can be imported into COMSOL, such as bifurcated pores. The etching process caused pores that were in close enough proximity to merge at their inlet, creating a single entrance orifice. (**B**) Fluidic simulation of **A**, showing higher velocity in the shallow bridge between pores as well as the faster velocity in the constrictions downstream. (**C**) Velocity surface plot of **A** superimposed with streamlines.

Section S6: Continuum Assumption for Simulations

Using the concentration of water in water and the volume of a single nanopore, we can perform a simple calculation to determine if the system is a continuum. Assuming that we have a 50 nm diameter pore that is 100 nm high, we can calculate the volume of the pore, making the basic assumption of the pore being cylindrical:

| $V= \pi r^{2}h$ | (5) |
| --- | --- |
| $V= \pi(2.5 \times{10}^{-8}m)^{2} \cdot1 \times{10}^{-7}m=1.96 \times{10}^{-22}m^{3}= 1.96 \times{10}^{-19} L$ | (6) |

which can then be used to determine the total number of particles within a single pore. The concentration of water in water is 55 M or 55 moles/liter. Multiplying by the volume of the pore, we can get the total number of moles of water inside the pore:

| $n=55 \frac{moles}{L} \cdot1.96 \times{10}^{-19} L= 1.08 \times{10}^{-17}moles of water$ | (7) |
| --- | --- |

and we can then multiply by Avogadro’s number to find the number of water molecules:

| $m= 1.08 \times{10}^{-17}moles \cdot6.022 \times{10}^{23}\frac{molecules}{mole}=6.5 \times{10}^{6} molecules/pore$ | (8) |
| --- | --- |

which corresponds to approximately 65,000 water molecules per 1 nm slice of the pore, thus the system can be defined as a continuum.

Section S7: Nanopore Sidewall Roughness Estimations

At the scale of the COMSOL simulations, we estimate the roughness we observe to correspond to atomic (~0.1 nm) or near-atomic (~1 nm) regime. However, our computational precision from the tomographic reconstructions (55-110 nm sections, ~1–2 nm/section) limits our extrapolation of the effects of these miniscule roughnesses on the bulk behavior within the pore. The reconstructed pore sidewall roughness can be estimated in ImageJ using a plugin (https://imagej.nih.gov/ij/plugins/roughness.html), cropping the tomogram (0.89 nm/pixel) to the sidewall projection. RMS roughness (*R*_q_) for these curves is estimated as *R*_q_ = 0.81 pixels = 0.72 nm. Additional noise is added when segmenting using similar intensity criteria and this is realized when using binary erode/dilate filters to fuse segmented regions. A sample z-projection of the segmented pore contours (20 nm, seen in Figure S2) where the contour is not narrowing appreciably shows ~5 nm maximal deviations across this range (0.89 nm/pixel). Using SEG3D2 software, it is possible to extract the points in cross-section contributing to a contour. Four line-sections were taken of pore sidewalls along XZ and YZ projections for smoothed and unsmoothed contours, and the RMS roughness (*R*_q_) was calculated based on a linear regression of the pore wall. For unsmoothed segmentations, *R*_q_ = 1.52 nm, *σ*_q_ = 0.53 nm. For smoothed contours, *R*_q_ = 1.05 nm , *σ*_q_ = 0.25 nm.


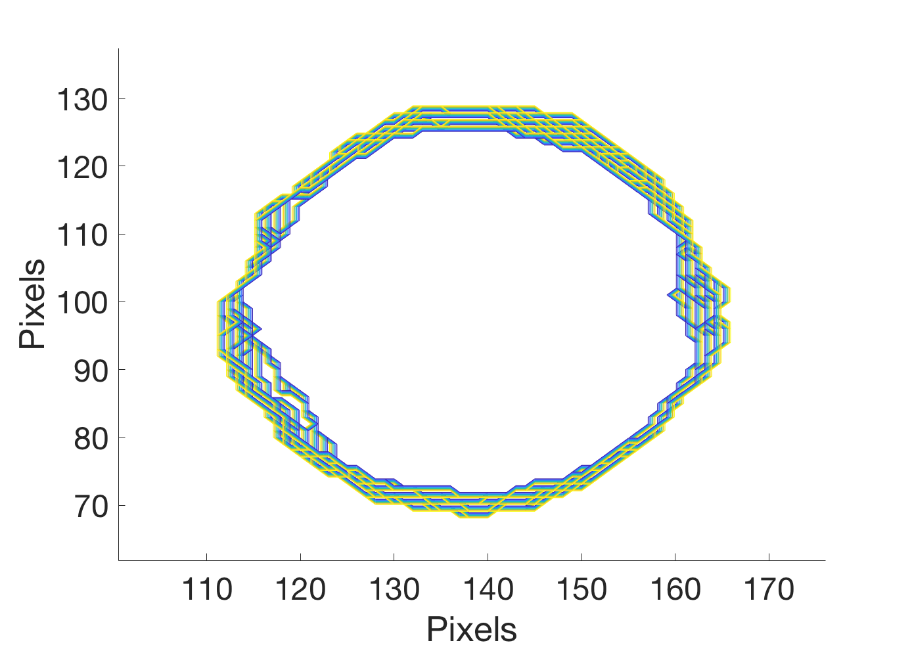


**Figure S7**: Overlaid 20 projected contours of a segmented nanopore
